# Comparison of Growth Characteristics and Genomics of Two Canine Distemper Virus Strains Isolated From Minks in China

**DOI:** 10.3389/fvets.2020.570277

**Published:** 2020-10-29

**Authors:** Rongshan Tao, Jie Chen, Tianyu Zhao, Chengyan Gong, Hongjun Pan, Rana Waseem Akhtar, Xue Li, Syed Aftab Hussain Shah, Qingjie Li, Jianjun Zhao

**Affiliations:** ^1^School of Public Health, The Key Laboratory of Environmental Pollution Monitoring and Disease Control, Ministry of Education, Guizhou Medical University, Guiyang, China; ^2^Division of Infectious Diseases, Institute of Special Animal and Plant Sciences, Chinese Academy of Agricultural Sciences, Changchun, China; ^3^School of Pharmacy, Changchun University of Traditional Chinese Medicine, Changchun, China; ^4^College of Animal Science and Technology, Jilin Agricultural University, Changchun, China; ^5^Department of Veterinary and Animal Sciences, Muhammad Nawaz Shareef University of Agriculture, Multan, Pakistan; ^6^Pakistan Scientific & Technological Information Centre (PASTIC), Quaid-i-Azam University Campus, Islamabad, Pakistan; ^7^Research Center of Traditional Chinese Medicine, The Affiliated Hospital to Changchun University of Chinses Medicine, Changchun, China; ^8^College of Animal Science and Veterinary Medicine, Heilongjiang Bayi Agricultural University, Daqing, China

**Keywords:** canine distemper virus, variant strains, complete genome, F protein, syncytia

## Abstract

Canine distemper (CD), caused by the CDV variant strain with H^I542N/Y549H^, has become an epidemic in fur-bearing animals in China since 2012. To well understand the genomic and replicated characteristics of the CDV variants, we determined the viral growth kinetics and completed the genome sequences of two CDV strains, namely SDZC(17)M2 and LNDL(17)M4, isolated from CDV-infected minks from Shandong and Liaoning province in China, in 2017. SDZC(17)M2 showed higher viral titers and extensive syncytia in BHK-minkSLAM (BMS) cells than LNDL(17)M4. Although both two strains belong to the Asia-1 genotype and clustered an independent clade in the phylogenetic tree, SDZC(17)M2, harboring I542N/Y549H substitutions in the H protein, shared high identity (99.3–99.6% nt) with the other variant strains, whereas LNDL(17)M4, with the only Y549H substitution, shared a lower identity (97.7%–97.9% nt) with the other variant strains. Furthermore, a novel R223K substitution was identified in the conserved cleavage site (RRQ*R*R → RRQ*K*R) of the F protein in the SDZC(17)M2 strain. However, it which did not significantly affect the cell to cell fusion activity when combined with the CDV H/minkSLAM in BHK-21 cells. The key variations in the genome contributed to the virulence and the evolutionary trend need to be determined in the future.

## Introduction

Canine distemper (CD) is a highly contagious, often fatal, multisystemic disease caused by canine distemper virus (CDV) in a wide range of mammalian hosts, with clinical symptoms including conjunctivitis, anorexia, diarrhea, lymphopenia, and encephalitis (1). CDV is an enveloped, single-stranded, negative-sense RNA virus belonging to the genus *Morbillivirus* within the family *Paramyxoviridae*. CDV particles are pleomorphic, frequently spherical, with a diameter ranging between 150 nm and 300 nm. Its genome comprises 15,690 nucleotides (nt) and encodes six structural and two non-structural proteins. The genomic RNA, tightly encapsulated by the nucleocapsid protein (N), is used as a template for transcription and replication, forming the ribonucleoprotein (RNP) together with the large protein (L) and phosphoprotein (P) (2). The RNP regulates the paramyxoviral genome replication and mRNA editing (3, 4). The P gene also encodes two non-structural proteins, C and V, which modulate the virulence and immune suppression activities of CDV by inhibiting the induction of the host's type I and II interferon (5). The L protein is an enzymatic component of the viral transcriptase and replicase (6), its gene is more conserved than other genes found in CDV (3). The matrix protein (M) has a significant impact on the budding of virus particles (6), while the major antigenic proteins, the haemagglutinin (H) and the fusion (F), can induce immune responses that protect the host from viral infection (7, 8). The H and F genes show high genetic variation and are used as suitable targets for the genotype identification and genetic analysis of the CDV strains (7, 8). The variations in H and F genes are considered as a possible cause underlying the higher occurrence of CDV infections reported in dogs (9). The H protein induces viral attachment to the host cells, whereas the F protein mediates the fusion between the viral envelope and host cell membrane (10). The 3′-untranslated region (3′-UTRs) and the 5′-untranslated region (5′-UTRs) specifically control transcription and replication (6). The UTRs between the M and F gene play an important role in modulating F protein expression and CDV virulence (11). CDV enters the susceptible hosts, by specifically recognizing and binding to the host's receptors, thus initiating the early stages of infection. To date, two molecules, namely signaling lymphocytic activation molecule (SLAM/CD150) and poliovirus receptor-related 4(Nectin4/PVRL4) have been identified as the cellular receptors of CDV (12, 13).

Since the outbreak in 2012, a number of fur-bearing animals (minks, foxes and raccoon dogs) have been infected with CDV variants, particularly with the I542N/Y549H substitutions of the H protein of CDV strains isolated in Shandong province in China, resulting in increased mortality and significant economic loss to the fur-bearing animal industry (14, 15). However, the characteristics of the CDV variant strains with the I542N/Y549H substitutions of the H protein remain unclear. In the present study, to better understand the characteristics of the CDV epidemic strains from fur-bearing animals, and we determined the viral growth kinetics and completed the genomic sequences of two CDV strains, namely SDZC(17)M2 and LNDL(17)M4, isolated from CDV infected minks in the Shandong and Liaoning provinces in China, respectively (16). Furthermore, the phylogenetic and genomic variations between these two strains and other CDV strains were analyzed. A novel R223K substitution in the conserved cleavage site (RRQ*R*R → RRQ*K*R) of F protein was identified in SDZC(17)M2. To further evaluate whether this substitution affects the interaction between CDV-H/F and mink Signaling Lymphocytic Activation Molecule (SLAM) we have constructed amino acids mutation expression plasmids, and conducted a cell to cell fusion assay. Our results could provide important clues to better understand variations of CDV strains and to further insights into the virus evolution.

## Materials and Methods

### Cells and Viruses

VeroDogSLAMtag (VDS) (Kindly gifted by Dr. Veronika von Messling, Paul-Ehrlich-Institute, Germany) cells expressing dog SLAM were cultured in Dulbecco's modified essential medium (DMEM, Wisent) supplemented with 5% heat-inactivated fetal bovine serum (FBS, Wisent). BHK-minkSLAM (BMS) cells expressing minkSLAM were established by our laboratory (17) and were cultured in DMEM supplemented with 5% heat-inactivated FBS (Wisent, Canada) and 1,000 μg/ml G418 (Sigma, USA). BHK-21 cells were purchased from ATCC and were cultured in DMEM (Wisent, Canada) supplemented with 5% heat-inactivated FBS (Wisent, Canada).

During July and October of 2017, minks from two fur-bearing animal farms in Shandong and Liaoning province, China, displayed severe depression, loss of appetite, dry nose, purulent secretion of the eye and nose, diarrhea, and foot thickening. Using the CD colloidal gold test strips (Kuailing, Shanghai, China), ill minks had been tested positive for CDV antigens. Finally, those minks in these farms had succumbed to CD despite receiving attenuated vaccines. Viral particles from these mink tissues (lung and spleen) were isolated using VeroDogSLAMtag (VDS) cells and identified using an indirect immunofluorescence assay (IFA) (16). In addition, the Hebei strain (GenBank accession no. KC427278) was isolated using Vero cells from a CDV-infected mink from Hebei in China in the year 2008 and maintained in our laboratory. The SD (14)7 strain (GenBank accession no. KP765763) and SD(14)11 strain (GenBank accession no. KP738610) were isolated using VDS cells from a CDV-infected fox and raccoon dog, respectively from Shandong in China in the year 2014, and maintained in our laboratory (18).

### Virus Growth Kinetics Analysis

BMS cells were plated in 6-well plates and infected with SDZC(17)M2, SD(14)7, SD(14)11 LNDL(17)M4, and Hebei strain at a multiplicity of infection (MOI) of 0.1 for 1 hour, and the inocula were replaced with DMEM. The cell-associated progeny virus was harvested daily for 6 days and the number of syncytia was counted under an inverted microscope. Virus titers were determined in VDS cells and expressed as TCID_50_ using Reed-Muench method. The assay was repeated 3 times and the mean value was obtained.

### RNA Extraction and RT-PCR

Total RNA was extracted from VDS cells infected with CDV SDZC(17)M2 and LNDL(17)M4 using a viral RNA extraction kit (Qiagen, Germany). Viral RNA was transcribed to cDNA using the PrimeScriptTMII 1st Strand cDNA Synthesis Kit (Takara, China) according to the manufacturer's instructions and the cDNAs were used for amplification of the full-length CDV genome using overlapping primers (Supplementary Table 1). PCR amplification was conducted using Phusion High-Fidelity DNA Polymerase (New England BioLabs). Briefly, 1.2 μL of the cDNA template was added to 48.8 μL of the RT-PCR master mix, including 0.4 μL each of the primer sets. The reaction conditions were as follows: initial denaturation at 94°C for 2 min, 35 cycles of denaturation at 94°C for 30 s, annealing at 50°C–60°C for 1 min, and elongation at 72°C for 1 min/kb, and a final extension at 72°C for 5 min. The PCR fragments were cloned into the pGEM-T vector (Promega, USA) and transfected into DH5α cells. The cloned plasmids were confirmed by sequencing using the Sanger method on an ABI 3730 sequencer (Comate Bio, Changchun, China) with the primers M13 F (5′-TGTAAAACGACGGCCAGT-3′) and M13 R (5′-CAGGAAACAGCTATGACC-3′).

### Phylogenetic and Genomic Sequence Analysis

The complete genome sequences of SDZC(17)M2 and LNDL(17)M4 were assembled after sequencing. The two isolated CDV strains were aligned by ClustalW along with CDV reference strains from the GenBank database. Phylogenetic trees were constructed with MrBayes version 3.2 using the maximum likelihood method. Each Bayesian tree was run for 5 million generations with a sample frequency of 1,000 generations. Tracer version 1.6 was used to determine whether the runs had reached stationarity and that a 25% burn was appropriate Ronquist. The tree was visualized using FigTree v.1.4.0.

To further explore the genetic characterization of SDZC(17)M2 and LNDL(17)M4 strains with seven CDV reference strains from Chinese fur-bearing animals, the complete nucleotide sequences of 5'UTR, 3'UTR, untranslated regions (N-P, P-M, M-F, F-H, H-L) and structural proteins (N, P, F, M, H, L) were analyzed. Among these representative CDV isolates, SY/raccoon dog/Jilin/2012/KJ466106, LN (10)1/fox/Liaoning/2010/KP765764, HLJ1-06/fox/Heilongjiang/2006/JX681125, and Hebei/mink/Hebei/2008/KC427278 were typical strains from four provinces, whereas SD(14)7/fox/2014/KP765763, SD(14)11/raccoon dog/2014/KP738610, and SD16F/fox/2016/MH337872 were identified as belonging to CDV H I542N/Y549H variant strains from Shandong province.

### Construction of F Gene Plasmids and Its Mutations

PCR amplification of SDZC(17)M2 and LNDL(17)M4 F genes were performed using the appropriate primers (Supplementary Table 2). The PCR-amplified F genes of SDZC(17)M2 and LNDL(17)M4 were cleaved with *Cpo*I, purified, and cloned into the mammalian expression vector pCI (Promega, USA), respectively named as pCI-SDZC(17)M2 Fwt and pCI-LNDL(17)M4 Fwt. Next, two single-site mutations of pCI-SDZC(17)M2 Fwt-K223R and pCI-LNDL(17)M4 Fwt-R223K were obtained using the QuikChange site-directed mutagenesis kit (Agilent, USA) with the relevant primers (Supplementary Table 2). All expression plasmids were verified using DNA sequencing.

### Cell Fusion Assay

To compare the differences in cell to cell fusion between different strains, a quantitative fusion assay was performed. Briefly, BHK-21 cells plated in 6-well plates were co-transfected with various expression plasmids: 0.5 μg of pCI-F, 1 μg pCI-H, and 0.5 μg pDisplay-minkSLAM using the X-tremeGENE HP DNA Transfection Reagent (Roche, Switzerland) according to the manufacturer's protocol. At 24 h post-transfection, the syncytia formation were observed using a microscope (10×).

To confirm the functional importance of CDV F protein, a quantitative fusion assay was performed as described previously (19), with slight modifications. One population of BHK-21 cells in a 6-well plate was co-transfected with 0.5 μg pCI-SDZC(17)M2 Fwt/pCI-SDZC(17)M2 Fwt-K223R or pCI-LNDL(17)M4 Fwt/pCI-LNDL(17)M4 Fwt-R223K, 1 μg of pCI-H expression plasmid or pCI empty vector, and 0.5 μg of the expression plasmid pCAG-T7 (encoding T7 DNA polymerase). In parallel, separate 6-well plates of BHK-21 cells were co-transfected with 1 μg of pDisplay-minkSLAM along with 0.5 μg luciferase gene under the control of the T7 promoter, and 0.02 μg renilla luciferase under the control of the thymidine kinase promoter (PRL-TK, Promega), Following an overnight incubation, both cell populations were mixed and incubated at 37°C, 5% CO_2_ for 5 hours. The cells were lysed using the Dual-Luciferase Assay System Lysis buffer (Promega, USA) for 20 min at 4°C. To quantify fusion, the relative fusion activity represented by the ratio of firefly luciferase activity to renilla luciferase activity was calculated. The luciferase or renilla activity were measured using a Dual-luciferase reporter Assay System (Glomax2020, Promega).

### Statistical Analyses

Statistical analyses were performed using the SPSS 17.0 package (SPSS Inc., Chicago, USA) and GraphPad Prism® 5 for Windows® (GraphPad software, San Diego., CA, USA). Significance was tested using one-way analysis of variance followed by Tukey's multiple comparisons test. *P* < 0.05, *P* < 0.01 and *P* < 0.001 were considered to indicate levels of statistical significance. The data were expressed as the mean ± S.E.M. from three independent experiments.

### Ethics Approval

No animal and human studies are presented in this manuscript and no potentially identifiable human images or data are shown.

## Results

### Growth Characteristics of CDV Strains in BHK-MinkSLAMtag (BMS) Cells

**Figure 1:**
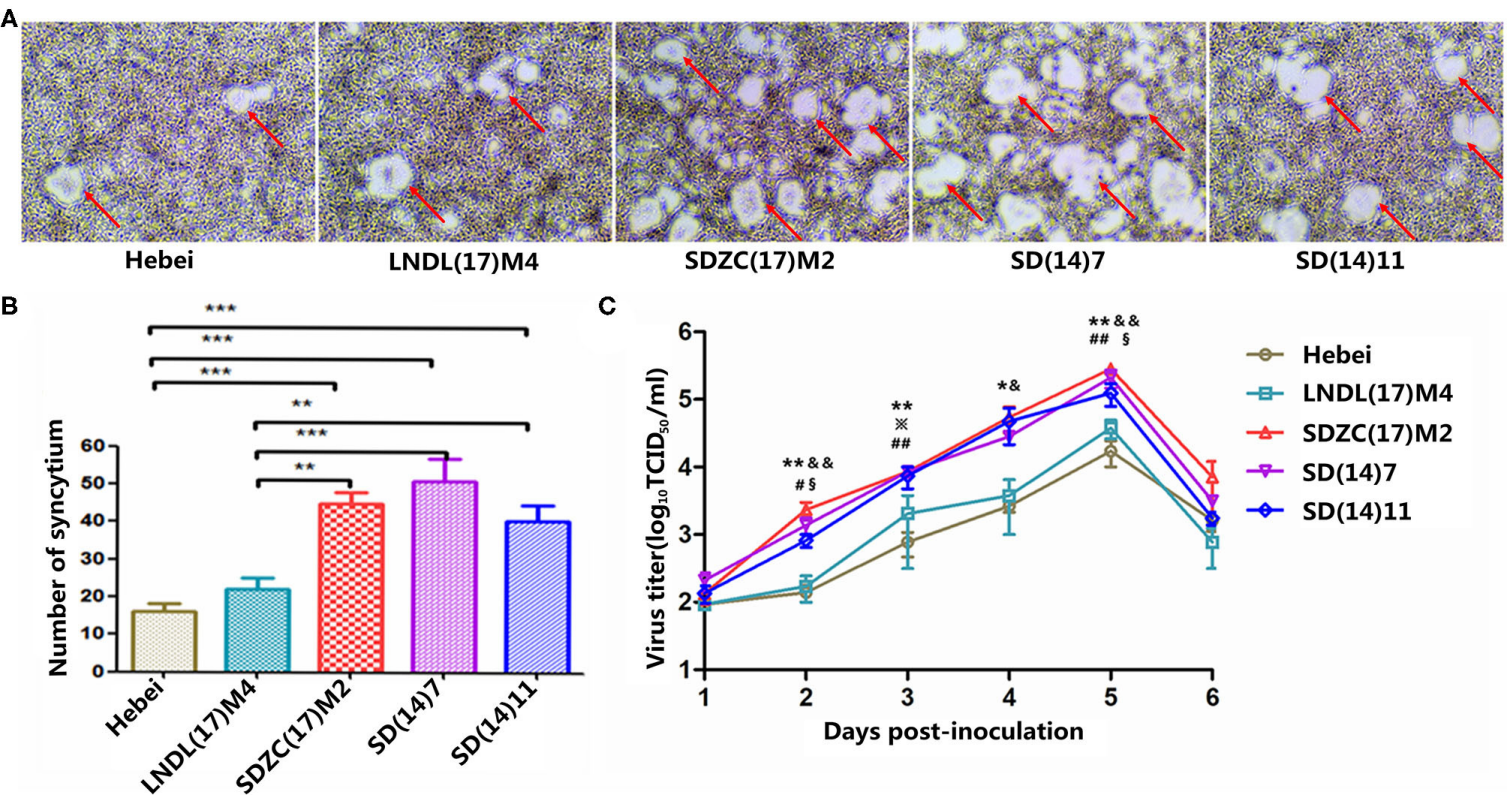
Characterization of CDV strains *in vitro*. **(A)** Cell fusion after 48 h of infection. **(B)** Statistical analysis of mean syncytial number. (*) indicates a significant difference between CDV strains (**P < 0.01; ***P < 0.001). **(C)** Cell-associated growth curves of SDZC(17)M2, SD (14)7, SD(14)11, LNDL(17)M4, and Hebei in BHK-mSLAM cells. BMS cells were infected with viruses at a multiplicity of infection of 0.1, and cell-associated progeny viruses were collected at the designated time points. The virus titer was determined using VDS cells using a limiting dilution method and the Reed-Muench method. (*) indicates a significant difference between Hebei and SDZC(17)M2 (*P < 0.05; **P < 0.01). (^#^) indicates a significant difference between Hebei and SD(14)7 (^#^*P* < 0.05; ^*##*^*P* < 0.01). (^※^) indicates a significant difference between Hebei and SD (14)11 (^※^*P* < 0.05). (^&^) indicates a significant difference between LNDL(17)M4 and SDZC(17)M2 (^&^*P* < 0.05; ^&&^*P* < 0.01). (^§^) indicates a significant difference between LNDL(17)M4 and SD(14)7 (^§§^*P* < 0.05).

To explore the differences in the growth characteristics of CDV strains, growth kinetic studies of five strains (Hebei, LNDL(17)M4, SDZC(17)M2, SD(14)7, and SD(14)11) were performed in BMS cells and the respective single step growth curves were determined. After 48 h post-infection, viruses induced syncytia, while their fusion-inducing capabilities varied (Figure 1A). LNDL(17)M4 and Hebei-infected BMS cells showed fewer syncytia, whereas the remaining three strains (SDZC(17)M2, SD(14)7, and SD(14)11) produced a more pronounced syncytia (Figure 1A). The number of syncytia of LNDL(17)M4-infected cells was significantly lower than that of SDZC(17)M2 (*P* < 0.01), SD(14)7 (*P* < 0.001), and SD(14)11 (*P* < 0.01), while the number of syncytia of SDZC(17)M2-infected cells was significantly higher than that of Hebei (*P* < 0.001) and LNDL(17)M4 (*P* < 0.01) (Figure 1B). Moreover, during the entire culture, the SDZC(17)M2 had higher titer than LNDL(17)M4 (*P* < 0.05, *P* < 0.01) and Hebei (*P* < 0.05, *P* < 0.01) (Figure 1C). The titer of the SDZC(17)M2, after 48 h post infection, was almost one order of magnitude higher than that of LNDL(17)M4 and Hebei (Figure 1C). The syncytia number and titers of SDZC(17)M2 and two strains (SD(14)7 and SD(14)11) were similar (Figure 1). No significant difference was observed between LNDL(17)M4 and Hebei, although the syncytia number and titers of LNDL(17)M4 was slightly higher (Figures 1B,C).

### CDV Complete Genome and Phylogenetic Analysis

To further explore the molecular characteristics of SDZC(17)M2 and LNDL(17)M4, the genomes of both CDV strains were amplified and sequenced. The complete genome sequences of CDV SDZC(17)M2 and LNDL(17)M4 strains were deposited in the GenBank database of NCBI with the accession number MK408454 and MK408453, respectively. Sequence analysis revealed that the SDZC(17)M2 genome shared the highest nucleotide identity (99.6%) with SD(14)7/fox/2014/KP765763 and SD(14)11/raccoon dog/2014/KP738610, and the lowest nucleotide identity (97.4%) with HLJ1-06/fox/Heilongjiang/2006/JX681125. In contrast, the LNDL(17)M4 genome shared the highest identity (99.1%) with SY/raccoon dog/Jilin/2012/KJ466106 and the lowest identity (97.7%) with the SDZC(17)M2 strain (Table 1). Furthermore, compared with the SDZC(17)M2 strain, other CDV variant strains were highly similar ranging from 98.8% to 100% at the amino acid level and 99.3%–99.6% at the nucleotide level. A comparison of nucleotide sequence with 9 CDV strains revealed similarities ranging from 97.4% to 99.8%, and a higher divergence of 2.6% was observed between variant strains (SD(14)7/fox/2014/KP765763, SD(14)11/raccoon dog/2014/KP738610, SD16F/fox/2016/MH337872, and SDZC(17)M2) and typical strains (SY/raccoon dog/Jilin/2012/KJ466106, LN(10)1/fox/Liaoning/2010/KP765764, HLJ1-06/fox/Heilongjiang/2006/JX681125, Hebei/ mink/Hebei/2008/KC427278 and LNDL(17)M4).

**Table 1:** Comparison of the full-length genomes of SDZC(17)M2 and LNDL(17)M4 to other representative CDV isolates (%).

**SDZC(17)M2**																
**Gene**	**SY**		**Hebei**		**LN(10)1**		**HLJ1-06**		**SD(14)7**		**SD(14)11**		**SD16F**		**LNDL(17)M4**	
	**aa**	**nt**	**aa**	**nt**	**aa**	**nt**	**aa**	**nt**	**aa**	**nt**	**aa**	**nt**	**aa**	**nt**	**aa**	**nt**
N	98.9	98.2	99	98.5	99.4	99.2	98.7	98.2	100	99.9	100	98.2	99.6	99.6	98.7	98.0
P	98	98.4	96.6	97.9	97.8	98.6	98.2	98.6	98.8	99.3	98.8	98.6	99.0	99.0	97.4	98.3
M	98.8	98.2	99.1	98.5	99.4	99.1	99.4	98.7	99.7	99.4	99.7	98.7	99.1	99.0	99.4	98.3
F	96.8	97.1	97.4	97.8	98.3	98.7	97.0	97.6	98.9	99.4	99.7	97.6	98.9	99.3	96.1	96.7
H	97.7	97.5	97.7	97.8	98.5	98.5	97.0	97.6	99.5	99.8	99.5	97.6	99.2	99.4	97.5	97.4
L	99.2	98.2	99.0	98.3	99.4	99.0	98.4	97.1	99.8	99.8	99.9	97.1	99.7	99.5	99.3	98.2
Total	-	97.8	-	97.9	-	98.7	-	97.4	-	99.6	-	99.6	-	99.3	-	97.7
**LNDL (17) M4**																
**Gene**	**SY**		**Hebei**		**LN(10)1**		**HLJ1-06**		**SD(14)7**		**SD(14)11**		**SD16F**		**SDZC(17)M2**	
	**aa**	**nt**	**aa**	**nt**	**aa**	**nt**	**aa**	**nt**	**aa**	**nt**	**aa**	**nt**	**aa**	**nt**	**aa**	**nt**
N	99.8	99.0	99.2	98.9	98.9	98.3	99.2	98.7	98.7	98.1	98.7	98.7	98.3	97.8	98.7	98.0
P	99	99.4	97.2	98.8	97.2	98.5	98.4	99.2	97.8	98.4	97.8	99.2	98	98.4	97.4	98.3
M	99.4	99.1	99.7	98.8	100	98.6	100	98.8	99.7	98.7	99.7	98.8	99.1	98.3	99.4	98.3
F	98.6	98.8	97.7	98.4	97.1	97.4	97.3	98.2	95.8	96.6	96.4	98.2	96.5	96.9	96.1	96.7
H	99.2	99.3	99.2	99.2	97.7	97.9	98.5	99.0	97.4	97.4	97.4	99.0	97.4	97.5	97.5	97.4
L	99.7	99.3	99.6	99.0	99.4	98.4	98.7	97.8	99.3	98.3	99.4	97.8	99.3	98.1	99.3	98.2
Total	-	99.1	-	98.9	-	98.1	-	98.2	-	97.9	-	97.8	-	97.8	-	97.7

To further examine the genomic variations between CDV variant and typical strains, their complete amino acid sequences and genome UTRs were analyzed in detail. A total of 16 amino acid substitutions and 11 nucleotide sites were identified between the translated region and UTR, respectively (Table 2). These substitutions were primarily distributed in the viral envelope protein coding genes such as F and H genes, as well as in the N, P, and L genes encoding the viral replication complex. We also compared the variant and typical strains of H protein and found the Y549H amino acid substitution in LNDL(17)M4, which was different from the typical strains (SY, LN(10)1, HLJ1-06, and Hebei), whereas the I542N amino acid substitution was not found.

**Table 2:** Amino acid and nucleotide sequences differences between the translated region and untranslated region (UTR) of typical (CDV SY, Hebei, LN(10)1, HLJ1-06, LNDL(17)M4) and variant strains (SD(14)11, SD(14)7, SD16F, SDZC(17)M2).

**Translated region**		
Gene	AA	typical strains → variant strains
N	2	Q442P, G507R
P	1	K98N (C)
M	1	G64S
F	4	N3S, I5T, K8N, R223K
H	3	P200S, M263I, I542N
L	5	H425Q, Y1169H, L1383V, L1585V, K1647Q
Total	16	
**Untranslated region**		
Gene	NT	typical strains → variant strains
3′UTR	-	-
N - P	1	G1704A
P - M	1	A3345G
M - F	4	C4501T, C4751T, G4803A, C4813A
F - H	2	T6986C, A7039G
H - L	1	G8936T
5′UTR	2	G15587A, T15613C
Total	11	

**Figure 2:**
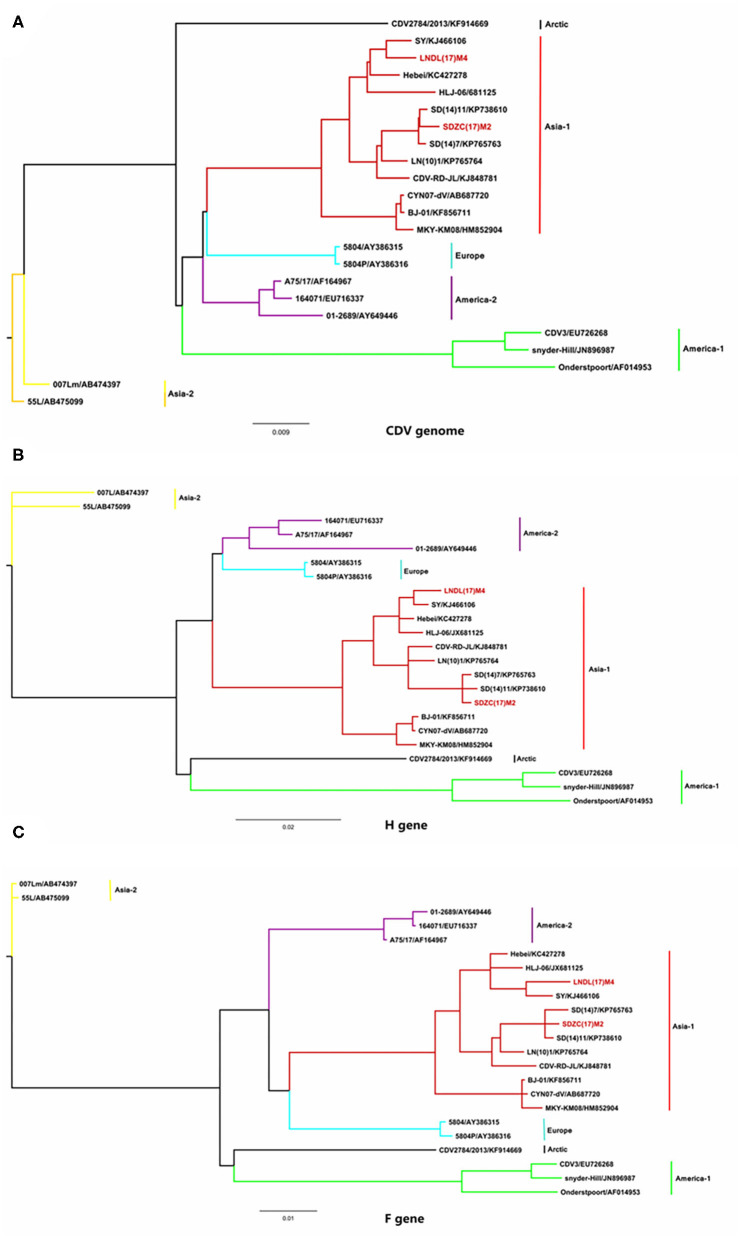
Phylogenetic analysis of CDV full-length gene sequences with 21 representative strains. **(A)** The phylogenetic analysis of CDV full-length gene sequence. **(B)** The phylogenetic analysis of CDV H gene sequence. **(C)** The phylogenetic analysis of CDV F gene sequence. The Bayesian phylogenetic tree was constructed based on the maximum likelihood method using MrBayes version 3.2 running for 5 million generations with a sample frequency of 1,000 generations. The red line represents the new isolate strains in the study.

To determine the genetic relationship between the SDZC(17)M2 and LNDL(17)M4 strains, the two CDV strains, along with 21 reference strains, were phylogenetically analyzed using the maximum likelihood method in MrBayes version 3.2. Based on the phylogenetic tree generated, 23 whole genome CDV isolates were divided into 6 genotypes: Asia-1, Asia-2, Europe, America-2, America-1, and Arctic (Figure 2A). The two new isolates, LNDL(17)M4 and SDZC(17)M2, belonged to Asia-1, which is a predominant genotype circulating in China (Figure 2B). The phylogenetic tree further revealed that SDZC(17)M2, SD(14)7, and SD(14)11 variant strains were more closely related and formed an independent clade (Figure 2B). The SDZC(17)M2 strain also clustered together with SD(14)7 and SD(14)11 strains, and were separated from the LNDL(17) M4 strain according to the analysis based on F and H sequences (Figures 2B,C). The results thus indicated a closer genetic relationship among CDV strains derived from the same geographical distribution.

### Amino Acid Sequence Analysis of the H and F Protein

**Figure 3:**
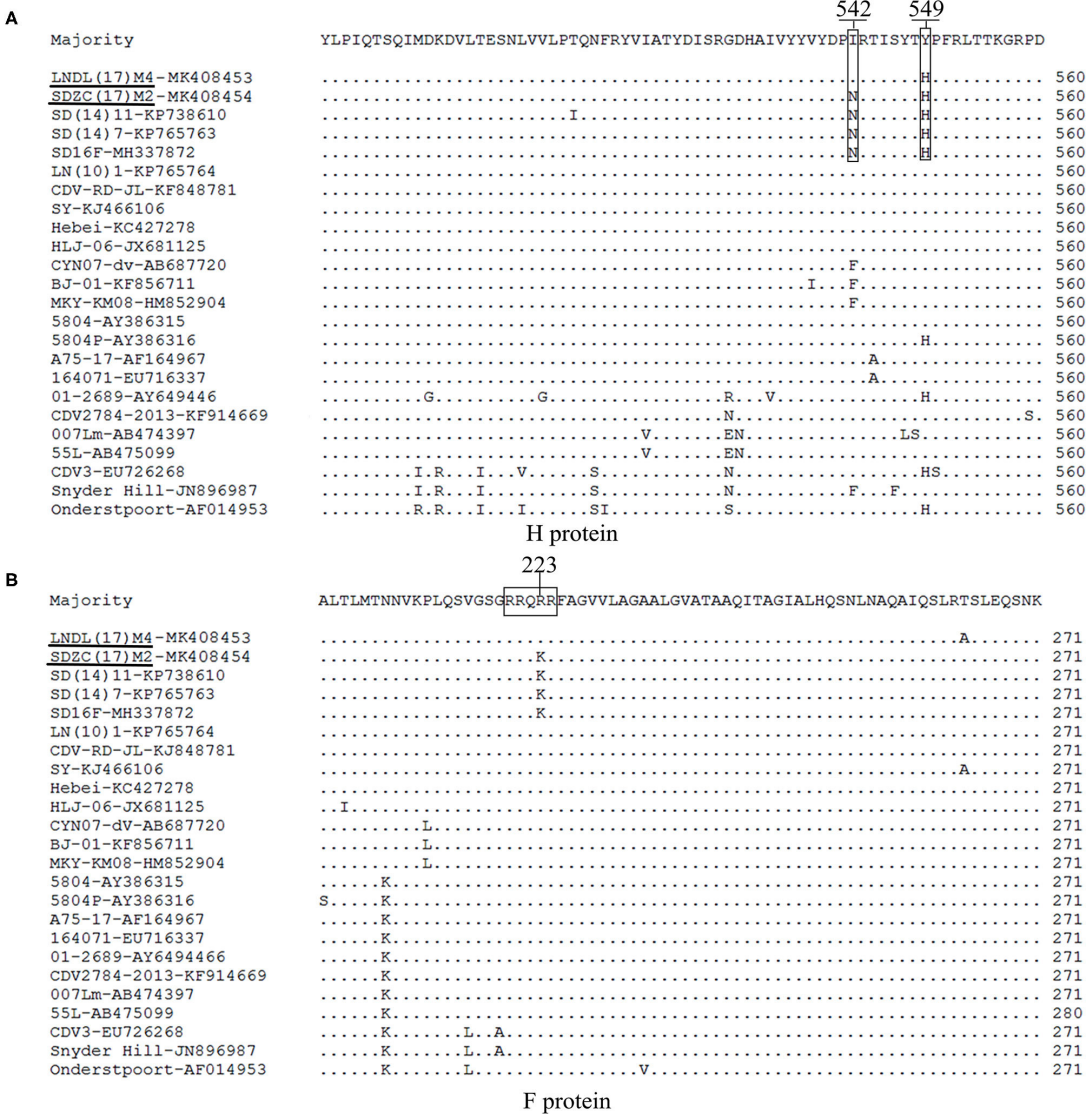
**(A)** The amino acid residues 542 and 549 in the H protein of LNDL(17)M4 and SDZC(17)M2 isolates were compared with other reference CDV isolates. The amino acid residues 542 and 549 are marked using boxes. The black line represents the new isolate strains in this study. **(B)** Amino acid sequence alignments of F protein from LNDL(17)M4 and SDZC(17)M2 with 30 reference strains. The cleavage site is indicated by the box.

The H amino acid sequences of the SDZC(17)M2 or LNDL(17)M4 isolates and other reference isolates were compared and analyzed (Figure 3). The isolates showed some unique molecular characteristics, including the amino acid N at position N542 and H549 (Figure 3A), which was also noticed to originate from fur-bearing animals in Shandong province from 2012 (15). Thus, we classified the SDZC(17)M2 isolate as a variant strain. We also discovered that the LNDL(17)M4 strain possessed the Y549H substitution in its H protein, which was found in fur-bearing animals as well as in observed from giant panda in China (20). The I542F substitution was also observed in the amino acid sequence of three Macaca mulatta-derived strains (KF856711, HM852904, and AB687720) and Snyder Hill strain (JN896987). The substitutions in H protein at the amino acid 542 and 549 positions seem to be closely related to the evolution of host selection and geographical distribution. Moreover, we found 9 potential *N*-glycosylation sites (19NSS, 149NFT, 309NGS, 391NQT, 422NIS, 456NGT, 584NIT, 587NST, and 603NRS) in the H amino acid sequences of LNDL(17)M4. However, SDZC(17)M2 had 10 *N*-glycosylation sites in its H amino acid sequences, which at position 542NRT, is different from the LNDL(17)M4 and LN(10)1, and Hebei strains isolated from the Liaoning and Hebei province, respectively. The novel *N*-glycosylation site at position 542-544 was reported in SDZC(17)M2, and was also found in other CDV strains (SD(12)3, SD(13)5, and SD(14)7) (14). However, our result confirmed that these I542N substitution was not a novel *N*-glycosylation site in the CDV H protein (manuscript in preparation).

**Figure 4:**
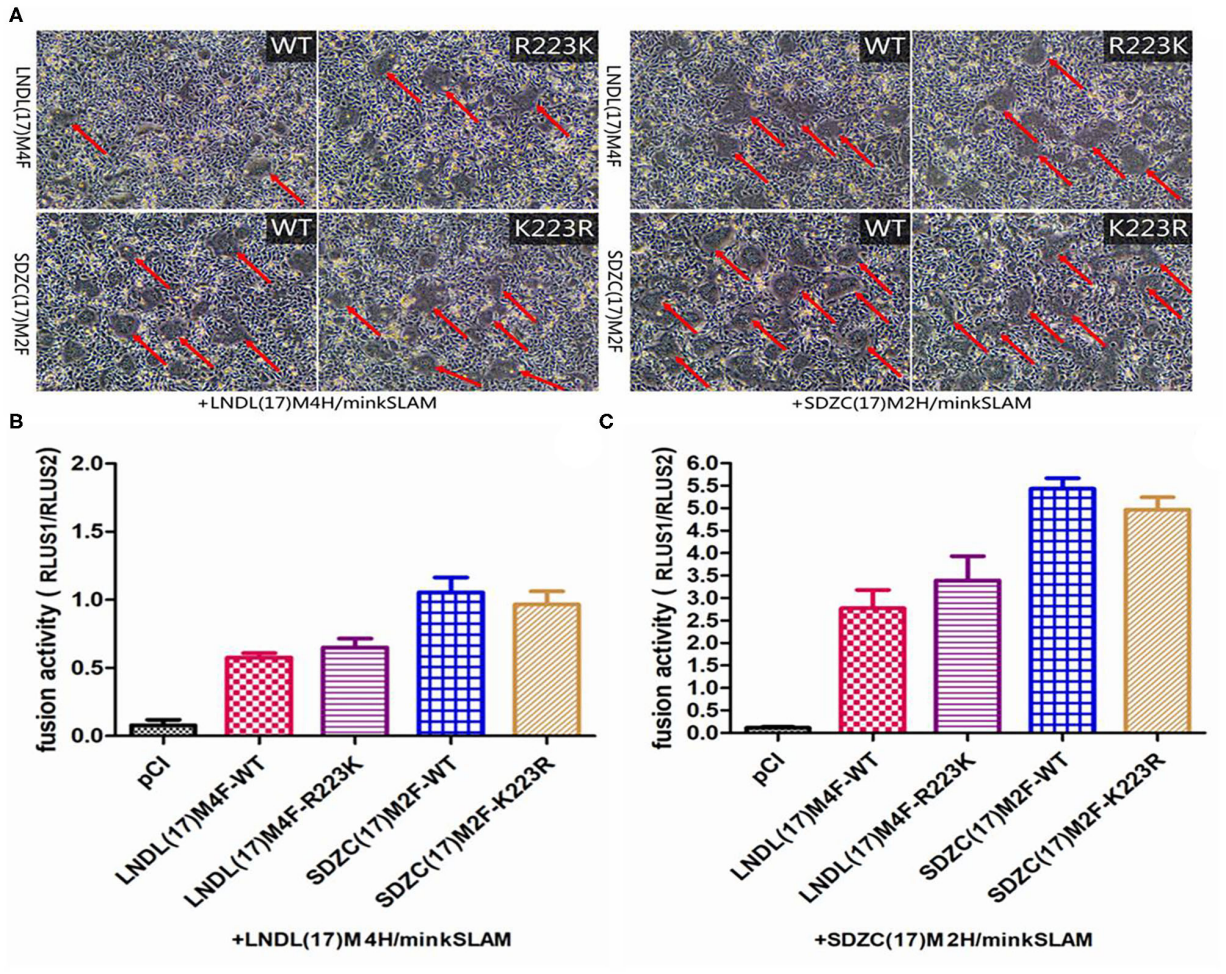
Cell fusion assay. **(A)** BHK-21 cells were co-transfected with the respective F, LNDL(17)M4H or SDZC(17)M2 H, and minkSLAM protein expression plasmids. Cell fusion was observed after 24 hours transfection. **(B)** CDV F or mutation-expressing plasmid fusion assay in BHK-21 cells co-transfected with H and minkSLAM. BHK-21 cells co-transfected with the plasmids for F, H, and T7 polymerases were co-cultured with BHK cells transfected with pT7-EMCluc, pRL-TK, and minkSLAM. The relative fusion activity was determined as the ratio of the firefly luciferase to Renilla luciferase activity after 5 hours. **(C)** CDV F or mutation-expressing plasmid fusion assay in BHK cells transfected with SDZC(17)M2H and minkSLAM. The relative fusion activity is shown as mean ± S.E.M of three independent experiments.

The cleavage sites in the C-terminus of the signal peptide (AQIHW) and in the F region (RRQRR) were highly conserved in all the CDV strains analyzed. However, the cleavage site (RRQKR) of SDZC(17)M2 showed an amino acid difference compared with other CDV stains, including the Asia-2 and vaccines strains. Interestingly, the cleavage site (RRQKR) substitution was also observed in the amino acid sequences of other CDV variant strains (Figure 4B). We then analyzed the potential *N*-glycosylation sites in the F protein and found 6 (62NRT, 108NAT, 141NLS, 173NVA, 179NCT, and 517NQS) sites in the LNDL(17)M4 and SDZC(17)M2 strains, of which four (141NLS, 173NVS, 179NCT, and 517NQS) were conserved in all CDV (Figure 3B). We also found a cysteine residue substitution C67R in LNDL(17)M4, whereas SDZC(17)M2 showed C116Y.

### Effect of the Novel R223K Substitution in F Protein Activity

To elucidate whether the R223K substitution of F protein affects the fusion activity, the pCI-SDZC(17)M2 and pCI-LNDL(17)M4 Fwt or their mutation-expressing plasmids were co-transfected with LNDL(17)M4H/minkSLAM or SDZC(17)M2H/minkSLAM in BHK-21 cells. The results showed that the CDV F R223K substitution resulted in cell fusion when combined with LNDL(17)M4H or SDZC(17)M2H (Figure 4A). To precisely determine the fusogenicity induced by the F R223K substitution, a luciferase assay was conducted, which showed that the novel R223K substitution of CDV F did not sufficiently affect the fusion activity compared with the Fwt when combined with LNDL(17)M4H or SDZC(17)M2H (Figures 4B,C). The pCI vector expression alone did not induce cell-cell fusion. Furthermore, compared with the typical strain LNDL(17)M4H, the variant strain SDZC(17)M2H had a higher luciferase activity when combined with Fwt or the mutation-expressing plasmids (*P* < 0.01, Figures 4B,C). The F R223K substitution did not substantially alter the fusion activity of CDV F protein. Therefore, we speculated that cell-cell fusion must be controlled by another factor.

## Discussion

In this study, we have compared the growth characteristics of LNDL(17)M4 and SDZC(17)M2 isolates with their respective similar strains *in vitro*. These strains induced syncytia formation with comparable sizes. Compared with the LNDL(17)M4 strain, the SDZC(17)M2 together with its similar SD(14)11 and SD(14)7 variant strains had higher virus titers and syncytia numbers in BMS cells (Figure 1). These results were consistent with the *in vivo* viral infections of minks, where we have demonstrated that SDZC(17)M2-infected minks displayed higher virulence index with 80% mortality than the LNDL(17)M4-infected minks (Manuscript in preparation). The SDZC(17)M2 variant strain had significant replication and spread capacity on cell lines expressing minkSLAM, whereas the adaptability of viruses in different species expressing SLAM/Nectin4 warrants further investigation.

The phylogenetic analysis showed that 23 strains could be divided into 6 genotypes based on the geographical distribution, and these CDV strains were isolated from different species such as foxes, raccoon dogs, minks, *M. mulatta*, and *Canis lupus familiaris*. Based on the F, H, and the CDV complete genome sequences, our results showed that the SDZC(17)M2 together with similar variant strains were more closely related to each other than to other CDV strains, which was consistent with the findings of Zhao et al. (14). The complete genome nucleotide sequence showed higher identity (99.3%–99.6% nt) between the SDZC(17)M2 isolate and other variant strains, whereas LNDL(17)M4 shared the lowest identity (97.7%–97.9% nt) with the variant strains (Table 1). In addition, a total of 16 amino acids in the coding region and 11 nucleotides in the non-coding region were different between the typical LNDL(17)M4 and variant SDZC(17)M2 isolates compared to their respective reference strains, which might be related to the increased replication efficiency of the SDZC(17)M2 strain (Table 2). In these amino acids, the 442 and 507 amino acid sites substitutions (Q442P, G507R) located in the C-terminal region of the N protein were associated with virus persistent infection during chronic inflammatory (21). The R223K substitution was also observed in helper T lymphocytes epitopes (212-283aa) on F protein (22). The 64 and 89 amino acid substitutions (P64S and E89K) in the M protein, which is contained in several vaccine strains, were responsible for the efficient growth of the recombinant virus on Vero cells (23).

Like all Paramyxoviruses, CDV F protein was synthesized as a F0 precursor and exhibits fusion activity only when cleaved into disulfide-linked F1 and F2 polypeptides (10). The consensus sequence of the CDV F protein cleavage site (RRQRR) was compared with other CDV strains. However, to the best of our knowledge, this is the first report of the R223K substitution in the conserved cleavage site (RRQKR) of the SDZC(17)M2-F protein (Figure 3). The virulence of Newcastle disease virus is primarily determined by the amino acid sequence surrounding the F protein cleavage site (24). However, the cleavage site of CDV is different from that of other members of Paramyxoviruses, such as measles virus (MV) (RRHKR), rinderpest virus (RPV) (RRHRR), dolphin Morbillivirus (DMV) (RRSKR), and petits ruminants (PPR) (RRTRR). We speculated that the cleavage site R223K substitution affected the functional conformation of F protein reacting with H protein, and changed the affinity between them causing further alterations in the efficiency of CDV-induced cell membrane fusion. We constructed CDV F mutants and conducted a series of experiments to verify this speculation. The results suggested that the R223K substitution in the F protein did not significantly change the relative fusion activity when combined with LNDL(17)M4H/minkSLAM or SDZC(17)M2H/minkSLAM in BHK-21 cells (Figure 4). Thus, the R223K substitution in the F protein may have no influence on virus virulence, but could be a compensatory mutation for changes at another position to counteract other residue alternation due to evolutionary forces. However, we found that CDV F and a series of other mutants efficiently bound SDZC(17)M2H, which significantly enhanced its relative fusion activity compared with LNDL(17)M4H. CDV H and F proteins play a key role in viral attachment and trigger sensitive cells syncytial formation (25), and the H protein is a major factor in determining the extent and efficiency of cell-cell fusion (26). The H protein had P200S, M263I, I542N, and Y549H substitutions between the typical and SDZC(17)M2 variant strains. Of these, we have shown that the Y549H and I542N/Y549H substitutions in the receptor-binding sites of the H protein significantly enhanced the level of cell membrane fusion in BMS cells (manuscript under preparation). In addition, some of the substitutions identified in this study have not been previously reported, indicating that these mutations were specific to the SDZC(17)M2 variant strain. Reverse genetics will help to determine whether a particular change or a combination of genetic changes (point mutations, insertions, and deletions) in CDV strains alter their viral infectivity, pathogenicity, and/or replication efficiency.

Herein, we have described the characteristics of two variant and preliminarily confirmed that the R223K substitution in F protein did not significantly change the relative cell-cell fusion activity in BHK-21 cells.

## Data Availability Statement

The datasets generated for this study can be found in online repositories. The names of the repository/repositories and accession number(s) can be found in the article/Supplementary Material.

## Ethics Statement

No animal and human studies are presented in this manuscript. No potentially identifiable human images or data is presented in this study.

## Author Contributions

QL and JZ conceived the study. RT, JC, TZ, CG, HP, RA, XL, SS, QL, and JZ were involved in all other aspects of the study, data collection, data analysis, and drafting and editing the paper. All authors have read and agreed to the published version of the manuscript.

## Conflict of Interest

The authors declare that the research was conducted in the absence of any commercial or financial relationships that could be construed as a potential conflict of interest.
