# Supplementary material for: Comparison of Growth Characteristics and Genomics of Two Canine Distemper Virus Strains Isolated From Minks in China
Source: Front Vet Sci. 2020 Oct 29;7:570277. doi: 10.3389/fvets.2020.570277 (PMC7658476; doi:10.3389/fvets.2020.570277)
Supplement: Supplementary file 1 [file Data_Sheet_1.doc]

**Supplementary Tables**

**Table S1. Primers used for amplification of complete genome sequences of LNDL (17) M4 and SDZC (17) M2**.

| **Primer** | **Primer sequence（5`-3`）** | **Position** | **Length** |
| --- | --- | --- | --- |
| **P1-F** | ACCAGACAAAGTTGGCTAAGGAT | 1-430bp | 430bp |
| **P1-R** | ATGCTTGGGATTACCTCTACTAACTTG |
| **P2-F** | GGAGCAATAAGAGGGATAAAG | 183-1631 | 1449bp |
| **P2-R** | GGTCCCAGGTTGACTGAGCAT |
| **P3-F** | GCTCAGCTAGTGTCAGAAATAG | 1278-1989 | 712bp |
| **P3-R** | TGATTCATCGAGATCCTGAGA |
| **P4-FSDZC(17)M2** | ATCCGACCACCCGTTCTATTC |
| **P4-R** | TCCAACACCTAAAGGCAGCGA |
| **P4-FLNDL(17)M4** | ATCCGACCAACCATTCTATCTCTAAATGGC | 1776-3674 | 1899bp |
| **P4-R** | CCCAACACCTAAAGGCAGTGATCCA |
| **P5-F** | AATGACTGAGGTGTACGACTTCG | 3431-4870 | 1440bp |
| **P5-R** | GCTTGCTACGTCCTGGACCCTAA |
| **P6-F** | AATTGTCTCGGTGTTGCTCCTGCA | 4532-5042 | 511bp |
| **P6-R** | TCGTCCTYGGGAGGTCYTGGTCTC |
| **P7-F** | GGACRTAGCAAGCCAACAG | 4858-7071 | 2214bp |
| **P7-R** | CTACCTGAGCCCTAAGTTTTC |
| **P8-F** | ACTTGAGAGGTTGGATGTAGG | 6629-7397 | 769bp |
| **P8-R** | ACCGTAACCCAATCTCATCTC |
| **P9-FLNDL(17)M4** | TTAGGGCTCAGGTAGTCCA |
| **P9-R** | CTAAGKCCAATTGARATGTGT |
| **P9-FSDZC(17)M2** | TTAGGGCTCAGGTAGTCCAACAATGC | 7058-8937 | 1880bp |
| **P9-R** | CTCAGTTCAATTGAGATGTATATCATCATACCATCA |
| **P10-F** | TACAACCAGTGTTGAGAATTTAGTC | 8842-10083 | 1242bp |
| **P10-R** | AGGAAAAGATCTCTCCTGTTATATG |
| **P11-F** | GCTCTAGACTTTGTTTTCATCACAG | 10026-11356 | 1331bp |
| **P11-R** | GTTTGGTTATCACCTTGGACAAGT |
| **P12-F** | AGGATTGCATCACTTGTCCAAGGT | 11322-12465 | 1144bp |
| **P12-R** | CCCACATGTGGTTCCTTAATGCT |
| **P13-F** | CCTAGAATAATAAACCGTTTG | 12318-13824 | 1507bp |
| **P13-R** | TATCCTCATCACTTTCGCATA |
| **P14-FLNDL(17)M4** | TTTGGGACAGTGGGATGATTG | 13653-15494 | 1842bp |
| **P14-R** | TTGGGATTGTCGTCAGGA |
| **P14-FSDZC(17)M2** | TTTTGGGACAGTGGGATGATTGA |
| **P14-R** | TGGGATTGTCGTCAGGATTAGAGACC |
| **P15-F** | ATGTTCCATGCATACCCAGTTT | 15267-15690 | 424bp |
| **P15-R** | ACCAGACAAAGCTGGGTATGAT |

**Table S2. Primers used for amplification of CDV F and its mutations gene**

| Primer | | Primer sequence（5’-3’） |
| --- | --- | --- |
|  | SDZC(17)M2-Fwt-F | atCGGACCGatgcacagcacaacccc |
|  | SDZC(17)M2-Fwt-R | atCGGTCCGtcaCTTATCGTCGTCATCCTTGTAATCgagtgatcttacatatgattttgaagttcc |
|  | LNDL(17)M4-Fwt-F | atCGGACCGatgcacaacaaaatccccaaa |
|  | LNDL(17)M4-Fwt-R | atCGGTCCGtcaCTTATCGTCGTCATCCTTGTAATCgagtgatcttacatatgatttcgaa |
|  | SDZC(17)M2-F K223R-F | cctgcaaaacgcctttgtctcctacctgaccctac |
|  | SDZC(17)M2-F K223R-R | gtagggtcaggtaggagacaaaggcgttttgcagg |
|  | LNDL(17)M4-F R223K-F | cctgcaaaacgcttttgtctcctacctgaccctac |
|  | LNDL(17)M4-F R223K-R | gtagggtcaggtaggagacaaaagcgttttgcagg |

The capital parts are enzyme sit. The underlined sites are FLAG label.
